# The Constitutive Proteome of Human Aqueous Humor and Race Specific Alterations

**DOI:** 10.3390/proteomes8040034

**Published:** 2020-11-18

**Authors:** Sai Karthik Kodeboyina, Tae Jin Lee, Lara Churchwell, Lane Ulrich, Kathryn Bollinger, David Bogorad, Amy Estes, Wenbo Zhi, Shruti Sharma, Ashok Sharma

**Affiliations:** 1 Center for Biotechnology and Genomic Medicine, Augusta University, Augusta, GA 30912, USA; SKODEBOYINA@augusta.edu (S.K.K.); TALEE@augusta.edu (T.J.L.); LCHURCHWELL@augusta.edu (L.C.); wzhi@augusta.edu (W.Z.); shsharma@augusta.edu (S.S.); 2 Department of Ophthalmology, Augusta University, Augusta, GA 30912, USA; LULRICH@augusta.edu (L.U.); kbollinger@augusta.edu (K.B.); DBOGORAD@augusta.edu (D.B.); AESTES@augusta.edu (A.E.); 3 Department of Population Health Sciences, Augusta University, Augusta, GA 30912, USA

**Keywords:** aqueous humor, mass spectrometry, proteomics

## Abstract

Aqueous humor (AH) is the fluid in the anterior and posterior chambers of the eye that contains proteins regulating ocular homeostasis. Analysis of aqueous humor proteome is challenging, mainly due to low sample volume and protein concentration. In this study, by utilizing state of the art technology, we performed Liquid-Chromatography Mass spectrometry (LC-MS/MS) analysis of 88 aqueous humor samples from subjects undergoing cataract surgery. A total of 2263 unique proteins were identified, which were sub-divided into four categories that were based on their detection in the number of samples: High (*n* = 152), Medium (*n* = 91), Low (*n* = 128), and Rare (*n* = 1892). A total of 243 proteins detected in at least 50% of the samples were considered as the constitutive proteome of human aqueous humor. The biological processes and pathways enriched in the AH proteins mainly include vesicle mediated transport, acute phase response signaling, LXR/RXR activation, complement system, and secretion. The enriched molecular functions are endopeptidase activity, and various binding functions, such as protein binding, lipid binding, and ion binding. Additionally, this study provides a novel insight into race specific differences in the AH proteome. A total of six proteins were upregulated, and five proteins were downregulated in African American subjects as compared to Caucasians.

## 1. Introduction

Aqueous humor (AH) is the fluid in the anterior and posterior chambers of the eye. It is produced by the non-pigmented ciliary body epithelium primarily through active transport of ions and solutes into the posterior chamber [1,2,3,4]. From the posterior chamber, the AH enters the anterior chamber via the lens and iris. After supporting the metabolic requirements of the avascular tissues of the anterior segment, the AH mainly exits the eye via the trabecular meshwork/Schlemm’s canal into the episcleral veins, known as conventional outflow. AH outflow also occurs via an alternative route through the ciliary muscle bundles into the supraciliary and suprachoroidal spaces, which is known as uveoscleral outflow [5].

AH is an integral component in many ocular health functions, including nutrient and oxygen supply, the removal of metabolic waste, ocular immunity, and ocular shape and refraction [6,7,8]. The dynamics of AH and the fine balance between production and drainage is essential in maintaining the physiological intraocular pressure (IOP) [2].

The major constituents of AH are water, electrolytes, organic solutes, cytokines, growth factors, and proteins [3,9,10,11]. The protein concentration in AH is in the range of 150 to 500 μg/mL [2]. Although proteins in AH are present in relatively low concentrations when compared to blood plasma, they are vital in the maintenance of anterior segment homeostasis [2,8,12,13,14,15,16,17,18,19]. Previous studies have shown significant alterations in several proteins in the AH obtained from eyes with glaucoma [12,13,14,15,20,21] and other eye disorders, including age-related macular degeneration [14,16,22,23,24,25].

Therefore, identifying the protein contents of AH is vital in understanding their physiological and pathological roles in the eye. However, given the low protein concentration and small volume, traditional low throughput approaches are not suitable for the proteomic analysis of AH. Liquid- chromatography Mass spectrometry (LC-MS/MS) has emerged as the analytical method of choice because of its high throughput nature, sensitivity, high dynamic range, and ability to identify complex mixtures even from small sample volumes [26,27]. However, many experimental and data-analytical hurdles exist, hampering the reliability and reproducibility of the data. In LC-MS/MS profiles, the proteins with high concentrations have a higher chance of detection, whereas the proteins with lower concentrations are detected only in a smaller percentage of samples due to random chance. It is exceedingly difficult to draw statistical conclusions for the proteins with a very low detection rate and should be excluded at the data analysis step. However, making such decisions that are based on the smaller sample set can lead to poor reproducibility. Therefore, a reference list of AH proteins detected reliably while using a larger sample set may be helpful in alleviating these concerns.

In this study, we identified the constitutive proteome of human aqueous humor, which may be useful as a reference for future studies, by utilizing a large sample set, state of the art technology, and revolutionary data analysis methods. Based on their abundance, the proteins were sub-divided into four (high, medium, low, and rare) categories. Interestingly, a comparison of African American and Caucasian subjects led us to the discovery of race-specific differences in the AH proteome, which are also presented in this study.

## 2. Materials and Methods

### 2.1. Human Subjects and Sample Collection

Aqueous humor samples were collected from 88 subjects undergoing cataract surgery at the Department of Ophthalmology, Medical College of Georgia at Augusta University. During these surgical procedures, a corneal incision is made, through which the aqueous humor fluid is evacuated from the anterior chamber and discarded. Instead of discarding, the AH samples were aspirated from the anterior chamber and collected in Eppendorf tubes. This method of sample collection is safe and efficient and it does not pose any risk to the subjects. The study was approved by the Institutional Review Board (IRB# 611480-13) at Augusta University, and written informed consent was obtained from all of the study participants. A chart review was conducted for all subjects to record their age, race, gender, smoking history, presence of systemic and ocular diseases, and IOP levels. Table 1 shows the characteristics of the study participants.

### 2.2. Aqueous Humor Sample Preparation

**Figure 1:**
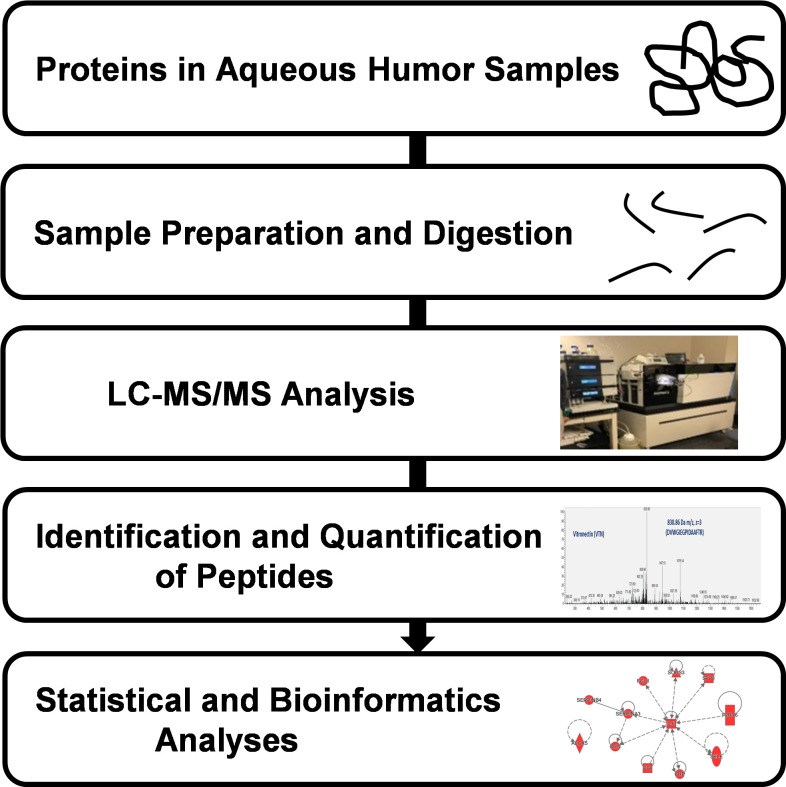
Liquid chromatography/Mass Spectrometry (LC-MS/MS) workflow for proteomic analysis of human aqueous humor. Samples were digested using trypsin and were analyzed using an Orbitrap Fusion Tribrid mass spectrometer coupled with an Ultimate 3000 nano-UPLC system. Proteins were identified and quantified using Proteome Discoverer (ver 1.4; Thermo Scientific, Waltham, MA, USA) followed by statistical analysis using the R Project for statistical computing (https://www.r-project.org/).

The aim of this study was to characterize all of the proteins present in the human aqueous humor and we did not utilize immunodepletion to remove abundant proteins. Aqueous humor samples (60 µL) were lyophilized and subsequently reconstituted in 30 µL of 8 M urea in 50 mM Tris-HCl (pH 8). 20 mM Dithiothreitol (DTT) was then added to the mixture in order to reduce cysteine residues, followed by alkylation with 55 mM iodoacetamide. 240 µL of 50 mM ammonium bicarbonate buffer was added in order to reduce urea concentration to below 1 M. Total protein concentration was measured while using a Bradford assay kit (Pierce, Rockford, IL, USA), according to the manufacturer’s instructions. The digestion of proteins was performed using a 1:20 ratio (*w*/*w*) of Trypsin (Pierce, Rockford, IL, USA) at 37 °C overnight. Figure 1 shows a schematic of the workflow involved in the AH sample preparation and proteomic quantification.

### 2.3. LC-MS/MS Analysis

The trypsin-digested samples were analyzed using an Orbitrap Fusion Tribrid mass spectrometer coupled with an Ultimate 3000 nano-UPLC system in order to perform in-depth proteomic characterization. Reconstituted peptides (6 μL) were trapped and washed on a Pepmap100 C18 trap at the rate of 20 μL/min using a gradient of 2% acetonitrile in water with 0.1% formic acid for 10 min. Subsequently, the peptide mixture was separated on a Pepmap100 RSCLC C18 column using a gradient of 2% to 40% acetonitrile with 0.1% formic acid for 120 min at a flow rate of 300 nL/min. Eluted peptides from the column were introduced into the Mass Spectrometer via nano-electrospray ionization source (temperature: 275 °C; spray voltage: 2000 V) and analyzed via data-dependent acquisition in positive mode. Orbitrap MS analyzer was used for precursor scan at 120,000 FWHM from 300 to 1500 *m*/*z*. MS/MS scans were taken while using an ion-trap MS analyzer in top speed mode (2-s cycle time) with dynamic exclusion settings (repeat count 1, repeat duration 15 s, and exclusion duration 30 s). Collision-induced dissociation (CID) was used as a fragmentation method with 30% normalized collision energy. The mass spectrometry proteomics data have been deposited to the ProteomeXchange Consortium via the PRIDE [28] partner repository with the dataset identifier PXD022463.

### 2.4. Protein Identification and Quantification

**Figure 2:**
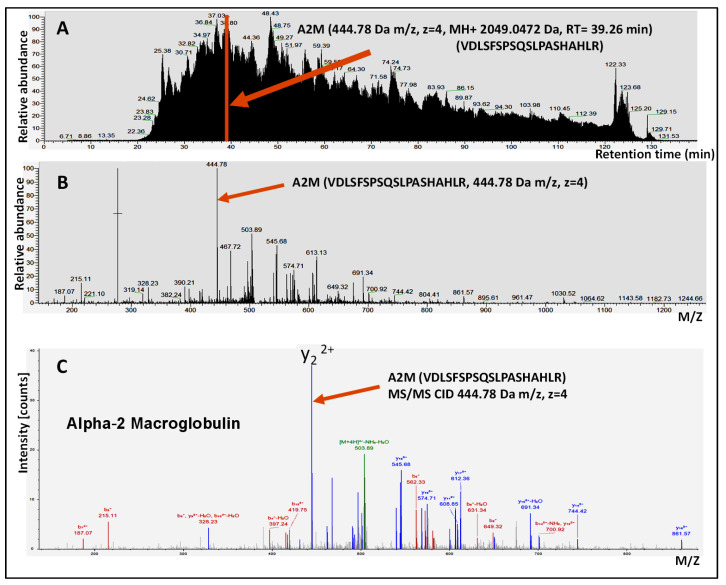
Example of LC-MS/MS analysis of human aqueous humor sample. (**A**) LC-MS/MS total ion current chromatogram. The retention time (RT) elution of one reporter peptide indicative of A2M protein is marked for illustration purposes. (**B**) MS spectra of selected precursor peptide 444.78 m/z with a distinct isotopic pattern benefitted from the high resolution of the Orbitrap MS analyzer. (**C**) MS/MS spectra using collision-induced dissociation (CID) fragmentation of A2M (444.78 *m*/*z*) precursor peptide, colored peaks (red for b ions and blue for y ions) indicate matches between experimental and theoretical/calculated values.

For the protein identification and quantification, raw MS data were processed using Proteome Discoverer software (ver 1.4; Thermo Scientific, Waltham, MA, USA) and then submitted for SequestHT search against the reviewed-manually annotated Uniprot- SwissProt human database with 20,385 entries. The following search parameters were used: 10 ppm precursor mass tolerance and 0.6 Da product ion tolerance; static carbidomethylation (+57.021 Da) for cysteine, dynamic oxidation (+15.995 Da) for methionine, and dynamic phosphorylation (+79.966 Da) for serine, threonine, and tyrosine. Proteins that contain similar peptides, which cannot be differentiated based on MS/MS analysis alone, were grouped in order to satisfy the principles of parsimony. A report comprising the identities and spectrum counts (number of peptide-spectrum match) for each protein was then exported as a semi-quantitative measure for relative protein levels that were detected in the AH sample. Figure 2 shows an example of LC-MS/MS analysis of one AH sample.

### 2.5. Statistical Analysis

The peptide-spectrum match (PSM) values from LC-MS/MS analysis were quantile normalized, and then log2 transformed to achieve normal distribution. For each protein, the detection rate (proportion of samples in which the protein was detected) was quantified. The proteins that were detected in a majority of samples (>50%) were examined in detail to see whether certain protein families were enriched in human AH. These commonly expressed proteins were also associated with gene ontology terms, including biological processes, cellular components, and molecular functions, using the “goana” function from “limma” (ver.3.40.6) R package. Adjusting for confounding variables, including age, sex and hypertension, differential expression analyses were performed using negative binomial regression, in order to discover differences in protein levels between African American and Caucasian subjects. The *p*-values were adjusted for multiple testing using the FDR method. All of the statistical analyses were performed using the R Project for Statistical Computing (version 3.5.1).

## 3. Results

### 3.1. Protein Content of the Human Aqueous Humor

**Figure 3:**
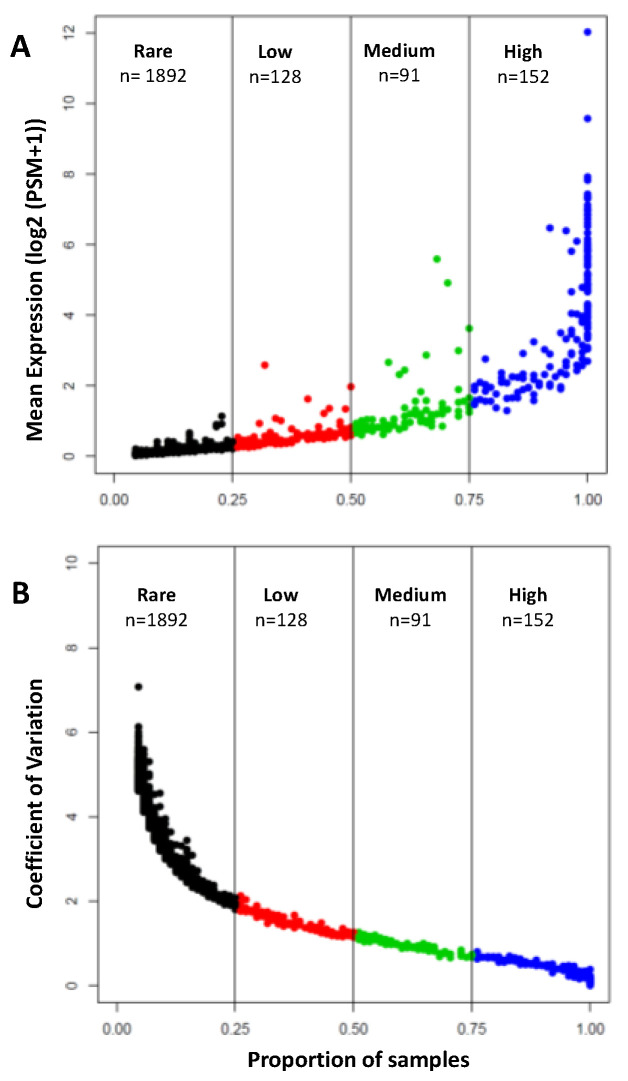
Distribution of the mean values (**A**) and coefficient of variation (**B**) of proteins detected in the human aqueous humor samples. The proteins were subdivided into four categories, based on their detection rate. High: detected in >75%; Medium: detected in 50–75%; Low: detected in 25–50%; Rare: detected in <25% of the samples. Coefficient of variation decreases as mean protein expression increases.

A total of 2263 unique proteins were identified in 88 aqueous humor samples (Table S1). These proteins were divided into four categories that were based on their detection in the number of samples: High (*n* = 152; detected in >75% of samples), Medium (*n* = 91; detected in 50–75% of samples), Low (*n* = 128; detected in 25–50% of samples), and Rare (*n* = 1892, detected in <25% of samples) (Figure 3A). Figure 3B shows the sample-to-sample variation in the levels of these proteins (the coefficient of variation). The majority of proteins in the “High” group show low sample-to-sample variation, indicating the uniformity of expression across samples. As the mean expression decreases, the coefficient of variation increases from high to rare proteins. Table 2 shows a complete list of 152 proteins found in at least 75% of AH samples.

### 3.2. Major Protein Families Detected in the Human Aqueous Humor

Five major protein families were found to be enriched in human aqueous humor, including Immunoglobulins (61 proteins), Complement proteins (25 proteins), Apolipoproteins (12 proteins), Serine Protease Inhibitors (16 proteins), and Insulin Growth Factor family (10 proteins). Table 3 lists all of the members of these five protein families detected in AH.

### 3.3. Gene Ontology Enrichment Analysis

**Figure 4:**
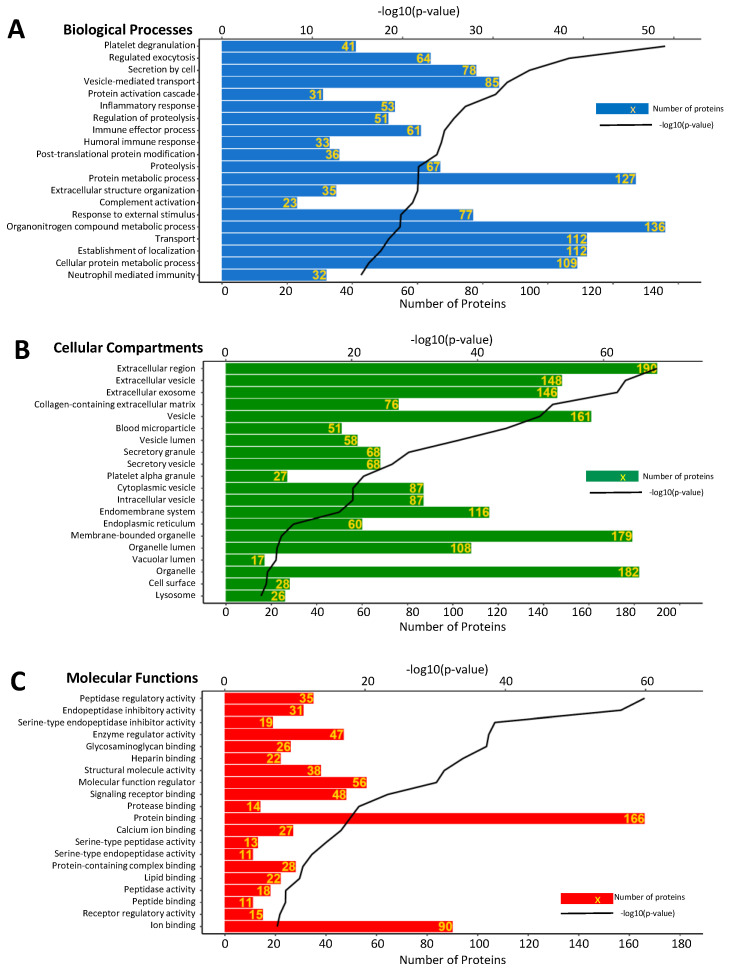
Biological processes (**A**), cellular components (**B**), and molecular functions (**C**) associated with the highly abundant aqueous humor proteins (detected in >50% samples). Bioinformatics analysis was performed in order to associate significantly enriched Gene Ontology (GO) terms to the constitutive aqueous humor proteome. The horizontal bars represent the number of proteins annotated to each GO term, and the black lines represent the *p*-value of enrichment.

A total of 243 proteins that were detected in at least 50% of the samples were considered as the constitutive proteome of human aqueous humor. Gene ontology enrichment analysis was performed in order to discover the biological processes, cellular components, and molecular functions associated with the constitutive proteome (Figure 4). The top enriched categories among the biological processes include organonitrogen metabolic process (136 proteins), protein metabolic process (127 proteins), transport (112 proteins), and establishment of localization (112 proteins). The most enriched cellular components are extracellular region (190 proteins), organelle (182 proteins), vesicle (161 proteins), extracellular vesicle (148), and extracellular exosome (146 proteins). Furthermore, protein binding (166 proteins), ion binding (90 proteins), molecular function regulator (56 proteins), signaling receptor binding (48 proteins), and enzyme regulator activity (47 proteins) were the top enriched molecular functions.

### 3.4. Network and Pathway Analysis

**Figure 5:**
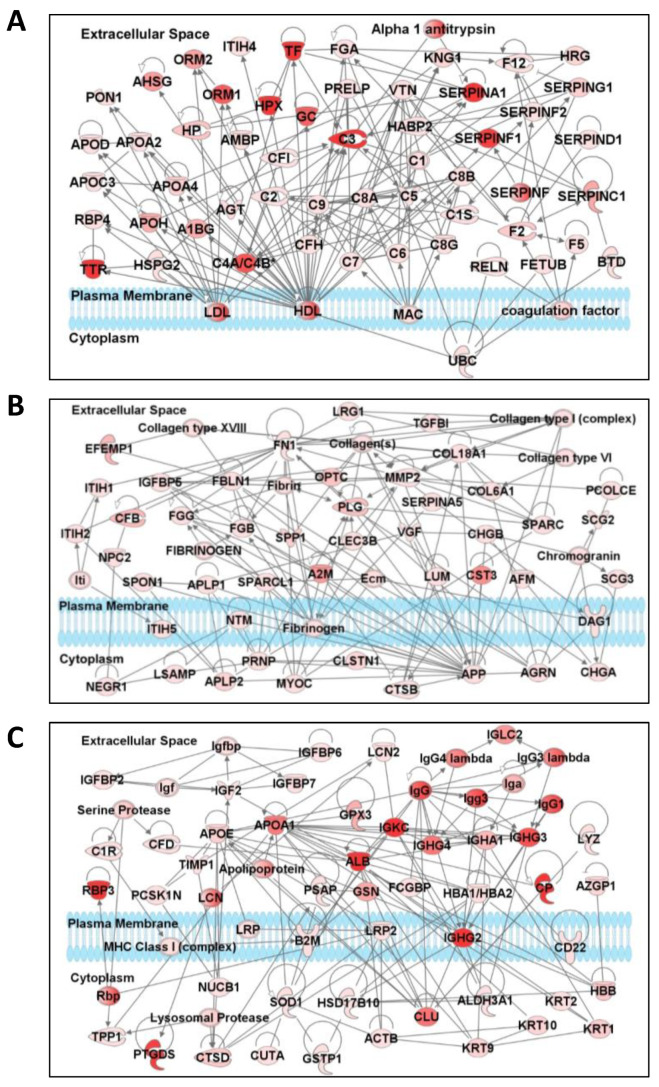
Three top-scoring interaction networks of highly abundant aqueous humor proteins. Ingenuity Pathway Analysis (IPA) was performed on the 243 proteins detected in at least half of the aqueous humor samples. (**A**) Network 1: includes several members of the Apolipoprotein, Complement, and SERPIN families. (**B**) Network 2: Network of proteins involved in tissue development, protein synthesis, and cellular compromise. (**C**) Network 3: Protein cluster associated with humoral immune response, inflammatory response, and protein synthesis. Each protein is represented as a node, and edges represent interactions between proteins. The intensity of color represents the relative levels of proteins (brighter red nodes indicate higher levels). Proteins are separated based on the cellular compartments.

Ingenuity Pathway Analysis (IPA) was used to discover the protein-protein interaction networks in the constitutive proteome (243 proteins) of human aqueous humor. Figure 5 presents the three top-scoring networks. Several members of the Apolipoprotein, Complement, and SERPIN families were part of the top-scoring network (Figure 5A). The second-highest scoring network consisted of 56 proteins, which are involved in tissue development, protein synthesis, and cellular compromise (Figure 5B). The third network includes several members of the Immunoglobulin and IGF families and other proteins that are involved in protein synthesis, humoral immune, and inflammatory responses (Figure 5C). IPA analysis also revealed that 21 canonical pathways were significantly enriched among the constitutive proteins observed in the AH (Table 4). The highly enriched canonical pathways include acute phase response signaling (40 proteins), LXR/RXR activation (33 proteins), FXR/RXR activation (32 proteins), clathrin-mediated endocytosis signaling (18 proteins), complement system (17 proteins), and coagulation system (14 proteins).

### 3.5. Aqueous Humor Proteins Associated with Race

**Figure 6:**
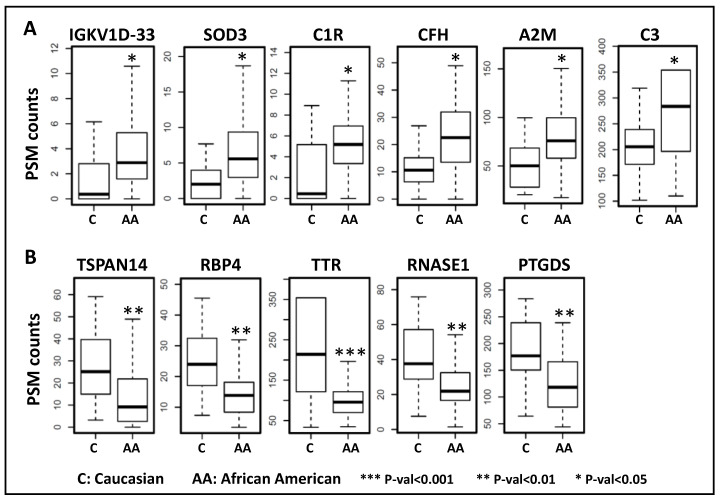
Race-specific differences in human aqueous humor proteins. A total of six proteins were upregulated (**A**) and five proteins were downregulated (**B**) in African American subjects as compared to Caucasian subjects.

Analyses were performed in order to discover race-specific differences in the AH proteome (differentially expressed in African Americans as compared to Caucasian subjects). A total of six proteins were upregulated and 5 proteins were downregulated in African American subjects (Table 5). Proteins significantly upregulated in African Americans subjects include Immunoglobulin kappa variable 1D-33 (IGKV1-33; FC = 2.191), Extracellular superoxide dismutase (SOD3; FC = 2.190), Complement C1r subcomponent (C1R; FC = 2.182), Complement Factor H (CFH; FC = 1.865), Alpha-2-macroglobulin (A2M; FC = 1.489), and Complement C3 (C3; FC = 1.289). The proteins significantly downregulated in African American subjects include Tetraspanin-14 (TSPAN14; FC = −2.089), Retinol-binding protein 4 (RBP4; FC = −1.753), Transthyretin (TTR; FC = −1.751), Ribonuclease pancreatic (RNASE1; FC = −1.636), and Prostaglandin D2 synthase (PTGDS; FC = −1.435). Figure 6 shows the boxplots depicting the distribution of these proteins in the African American and Caucasian subjects.

## 4. Discussion

This study provides the proteomic repertoire of human AH while using a larger sample set and highly sensitive mass spectrometry technology. The low abundant proteins have higher variation and poor reproducibility due to random nature of detection of proteins in mass spectrometry analysis. This study provides a reference AH proteome, which can be used in order to enhance the interpretation of results in future studies. We identified 243 proteins in at least 50% of samples, which we refer to as the constitutive proteome of human aqueous humor.

A comparison of our study with a previously published study by Chowdhury et. al. [2] revealed significant overlap in the proteins identified in human AH. We detected more than 79% of the 355 AH proteins that were identified in the previous study using nano-LC-ESI-MS/MS. Also, in the previous study, the samples were divided into three matched groups and 206 proteins were found in all three groups. A comparison of these 206 proteins with the constitutive AH proteome of our study (243 proteins) revealed >70% overlap [2].

Our comprehensive proteomic analysis revealed that five protein families are highly enriched in human aqueous humor, including apolipoproteins, complement proteins, immunoglobulins, IGF family proteins, and serine protease inhibitors (SERPINs). Apolipoproteins are proteins that bind and transport lipids in biological fluids. Seven apolipoproteins, including APOA1, APOA2, APOA4, APOC3, APOD, APOE, and APOH, were highly abundant, whereas five apolipoproteins APOB, APOC1, APOLD1, APOF, and APOL1 were detected in less than 25% of samples. Consistent to our findings, APOA1, APOA2, APOA4, APOD, APOE, and APOH were also identified in previous studies [2,29,30]. Several members of this family were part of the top scoring protein interaction network identified while using IPA analysis.

The anterior chamber is immune privileged and relies on AH to maintain a pathogen-free environment. Our analysis identified 25 complement proteins from both the classical and alternative pathways. Eleven complement proteins, including CFI, C4B, C6, C8A, and C9, were detected in more than 75% of the samples. Similar to the blood plasma, several members of the Immunoglobulin family of proteins were also identified in the AH. Immunoglobulins are involved in cell communication, defense response, and the regulation of metabolic processes. The presence of a wide array of immunoglobulins has been reported in previous studies indicating their existence in the AH of cataract and glaucoma patients [12,31,32].

Insulin-like growth factors and their binding proteins have been shown to play an important role in ocular functions. Ten IGF family proteins were identified in our analyses. Several IGFBPs in vitreous and aqueous humor have been previously reported [33]. However, the predominant serum carrier protein, IGFBP3, was present in less than 50% of AH samples, whereas IGFBP7 and IGFBP6 were highly abundant, indicating quantitative differences between the two fluids. IGFBP7 has been linked to hypertensive retinopathy and familial retinal macroaneurysms, indicating its role in retinal vascular pathology [34,35]. Furthermore, IGFBP7 was elevated in the AH of exudative age-related macular degenerative patients and is considered to be an anti-angiogenic agent in these patients [36].

The SERPIN family of proteins are ubiquitous in the body and their abnormalities are associated with ‘serpinopathies’. Ten proteins of this family, including SERPINC1, SERPINF1, SERPINA3, SERPINA1, SERPING1, SERPINF2, SERPIND1, SERPINA6, SERPINA4, and SERPINI1 were detected in at least 75% of samples. SERPINA3 is an acute phase response protein, which is involved in retinal angiogenesis and inflammation [37,38]. SERPINC1 or Antithrombin III deficiency has been associated with retinal vein occlusion, consistent with its role as an anti-clotting agent [39]. A decrease in SERPINF1 was associated with neovascularization and high myopia [40,41]. Overall, this glycoprotein is known to possess beneficial effects, such as potent anti-angiogenic, anti-thrombotic, anti-tumorigenic, anti-inflammatory, and neuroprotective properties [40,41,42,43,44,45,46,47]. SERPING1, which is the largest member of the superfamily, has been associated with suppressing inflammatory conditions, fibrinolysis and blood coagulation [48,49].

Interestingly, after adjusting for confounding variables, including age, sex, and hypertension, we found 11 proteins that were differentially expressed between African American and Caucasian subjects, indicating race-specific differences in the AH proteome. Overall, six proteins were significantly upregulated, while five proteins were downregulated in African American subjects. Proteins related to immune responses such as IGKV1D-33, C6, C8A were present at elevated levels in African American subjects. Among the downregulated proteins, TSPAN-14 was at least two-fold lower in African Americans. A member of this family, TSPAN-12, was discovered as a therapeutic target for retinal vascular diseases, such as age-related macular degeneration and diabetic retinopathy [50]. Three other vision-related proteins, including RBP4, TTR, and PTGDS, were present in lower levels in the African American population. RBP4, a retinol transporter protein, is known to be involved in congenital eye disease [51]. TTR is a transport protein, which carries retinol-binding protein and is essential for the maintenance of photoreceptors, visual cycle, and perception [52]. PTGDS is a secretory retinoid transporter that is involved in the maintenance of the blood-retinal barrier. The difference in the vision-related proteins might be one of the contributing factors for increased risk of eye-related ailments in the African American population.

## 5. Conclusions

In conclusion, this study characterized the human aqueous humor proteome using the latest technology and a larger sample set. A total of 243 proteins, which were detected in at least half of the samples, were considered to be the constitutive proteome of human aqueous humor. Five protein families were highly enriched in the human aqueous humor proteome. Eleven proteins were significantly altered between African American and Caucasian subjects, indicating race-specific differences. The highly abundant aqueous humor proteins are involved in immune-mediated responses, transport, metabolism, and binding. The reliable characterization of the aqueous humor proteome will provide new insights into the factors that govern anterior segment homeostasis and aid in biomarker discovery in various eye disorders.

## Figures and Tables

**Table 1:** Characteristics of study participants.

Characteristics	Count
Subjects, (*n*)	88
Sex: F/M	55/33
Age (years)	67.0 ± 9.56
Race: AA/Caucasian	66/22
Hypertension, N/Y	41/47
Smoking, N/Y	56/32
Cardiovascular disease, N/Y	81/7
Cerebrovascular disease, N/Y	87/1
Collagen vascular disease, N/Y	75/13
Intraocular Pressure (IOP)	19.5 ± 7.01

**Table 2:** Most abundant proteins present in the human aqueous humor.

Uniprot ID	Gene Symbol	Description	Detected in Proportion of Samples (%)	Mean PSM Value
P02768	ALB	Albumin	100.00	4202.61
P02787	TF	Serotransferrin	100.00	765.31
P01024	C3	Complement C3	100.00	255.00
P01009	SERPINA1	Alpha-1-antitrypsin	100.00	233.24
P02790	HPX	Hemopexin	100.00	176.64
P01859	IGHG2	Immunoglobulin heavy constant gamma 2	100.00	168.92
P10745	RBP3	Retinol-binding protein 3	100.00	166.25
P00450	CP	Ceruloplasmin	100.00	165.20
P01834	IGKC	Immunoglobulin kappa constant	100.00	161.42
P02766	TTR	Transthyretin	100.00	156.30
P41222	PTGDS	Prostaglandin-H2 D-isomerase	100.00	142.25
P36955	SERPINF1	Pigment epithelium-derived factor	100.00	141.33
P01860	IGHG3	Immunoglobulin heavy constant gamma 3	100.00	127.65
P02763	ORM1	Alpha-1-acid glycoprotein 1	100.00	119.16
P02774	GC	Vitamin D-binding protein	100.00	115.89
P0DOX7	N/A	Immunoglobulin kappa light chain	100.00	108.22
P10909	CLU	Clusterin	100.00	102.33
P02647	APOA1	Apolipoprotein A-I	100.00	96.78
P01034	CST3	Cystatin-C	100.00	82.73
P02765	AHSG	Alpha-2-HS-glycoprotein	100.00	73.27
P01023	A2M	Alpha-2-macroglobulin	100.00	72.77
P19652	ORM2	Alpha-1-acid glycoprotein 2	100.00	68.70
P06396	GSN	Gelsolin	100.00	62.66
P01008	SERPINC1	Antithrombin-III	100.00	62.65
P02749	APOH	Beta-2-glycoprotein 1	100.00	59.09
P04217	A1BG	Alpha-1B-glycoprotein	100.00	59.04
P22352	GPX3	Glutathione peroxidase 3	100.00	58.23
Q9UBP4	DKK3	Dickkopf-related protein 3	100.00	56.91
P01011	SERPINA3	Alpha-1-antichymotrypsin	100.00	53.15
P01876	IGHA1	Immunoglobulin heavy constant alpha 1	100.00	51.83
Q12805	EFEMP1	EGF-containing fibulin-like extracellular matrix protein 1	100.00	50.79
P00751	CFB	Complement factor B	100.00	50.35
Q13822	ENPP2	Ectonucleotide pyrophosphatase/phosphodiesterase family member 2	100.00	45.77
P00747	PLG	Plasminogen	100.00	45.45
Q9UBM4	OPTC	Opticin	100.00	43.07
P07339	CTSD	Cathepsin D	100.00	39.10
P00734	F2	Prothrombin	100.00	38.21
P05155	SERPING1	Plasma protease C1 inhibitor	100.00	35.94
P10451	SPP1	Osteopontin	100.00	35.94
P06727	APOA4	Apolipoprotein A-IV	100.00	33.93
P02649	APOE	Apolipoprotein E	100.00	32.43
P01042	KNG1	Kininogen-1	100.00	30.10
P04196	HRG	Histidine-rich glycoprotein	100.00	29.90
P25311	AZGP1	Zinc-alpha-2-glycoprotein	100.00	29.67
P07998	RNASE1	Ribonuclease pancreatic	100.00	29.30
O94985	CLSTN1	Calsyntenin-1	100.00	27.54
P01019	AGT	Angiotensinogen	100.00	26.00
P02760	AMBP	Protein AMBP	100.00	24.23
P02652	APOA2	Apolipoprotein A-II	100.00	20.83
P36222	CHI3L1	Chitinase-3-like protein 1	100.00	20.03
P23142	FBLN1	Fibulin-1	100.00	19.91
P02750	LRG1	Leucine-rich alpha-2-glycoprotein	100.00	18.87
P05156	CFI	Complement factor I	100.00	18.82
P02753	RBP4	Retinol-binding protein 4	100.00	17.09
P04004	VTN	Vitronectin	100.00	16.58
Q16270	IGFBP7	Insulin-like growth factor-binding protein 7	100.00	15.10
P51884	LUM	Lumican	100.00	13.89
P00746	CFD	Complement factor D	100.00	13.10
P05090	APOD	Apolipoprotein D	100.00	12.66
O43505	B4GAT1	Beta-1,4-glucuronyltransferase 1	100.00	12.09
P24592	IGFBP6	Insulin-like growth factor-binding protein 6	100.00	11.28
Q96PD5	PGLYRP2	N-acetylmuramoyl-L-alanine amidase	100.00	11.19
Q14515	SPARCL1	SPARC-like protein 1	100.00	11.01
P61916	NPC2	NPC intracellular cholesterol transporter 2	100.00	9.57
Q99969	RARRES2	Retinoic acid receptor responder protein 2	100.00	8.03
P61769	B2M	Beta-2-microglobulin	100.00	7.77
Q99714	HSD17B10	3-hydroxyacyl-CoA dehydrogenase type-2	100.00	7.61
Q06481	APLP2	Amyloid-like protein 2	98.86	30.95
P43652	AFM	Afamin	98.86	17.18
Q8IZJ3	CPAMD8	C3 and PZP-like alpha-2-macroglobulin domain-containing protein 8	98.86	17.14
Q14767	LTBP2	Latent-transforming growth factor beta-binding protein 2	98.86	15.82
Q15582	TGFBI	Transforming growth factor-beta-induced protein ig-h3	98.86	14.68
Q9Y5W5	WIF1	Wnt inhibitory factor 1	98.86	14.39
Q08629	SPOCK1	Testican-1	98.86	8.93
P07602	PSAP	Prosaposin	98.86	7.48
Q08380	LGALS3BP	Galectin-3-binding protein	98.86	6.67
P00748	F12	Coagulation factor XII	98.86	6.06
P0DOY2	IGLC2	Immunoglobulin lambda constant 2	97.73	75.13
P16870	CPE	Carboxypeptidase E	97.73	18.17
Q9HCB6	SPON1	Spondin-1	97.73	10.63
P06312	IGKV4-1	Immunoglobulin kappa variable 4-1	97.73	6.06
P51888	PRELP	Prolargin	97.73	5.19
P0DOX8	N/A	Immunoglobulin lambda-1 light chain	96.59	64.10
P00738	HP	Haptoglobin	96.59	43.35
P08603	CFH	Complement factor H	96.59	20.12
Q8NG11	TSPAN14	Tetraspanin-14	96.59	17.09
Q14624	ITIH4	Inter-alpha-trypsin inhibitor heavy chain H4	96.59	14.89
Q99972	MYOC	Myocilin	96.59	8.76
Q9NQ79	CRTAC1	Cartilage acidic protein 1	96.59	8.24
P01861	IGHG4	Immunoglobulin heavy constant gamma 4	95.45	99.95
P35555	FBN1	Fibrillin-1	95.45	12.27
Q9BSG5	RTBDN	Retbindin	95.45	6.64
P05452	CLEC3B	Tetranectin	95.45	6.41
Q92520	FAM3C	Protein FAM3C	95.45	5.67
Q86UP8	GTF2IRD2	General transcription factor II-I repeat domain-containing protein 2A	95.45	5.51
P02748	C9	Complement component C9	94.32	13.93
O14773	TPP1	Tripeptidyl-peptidase 1	94.32	5.24
P01033	TIMP1	Metalloproteinase inhibitor 1	94.32	3.64
Q15113	PCOLCE	Procollagen C-endopeptidase enhancer 1	94.32	3.45
P01700	IGLV1-47	Immunoglobulin lambda variable 1-47	93.18	4.61
P0C0L5	C4B	Complement C4-B	92.05	124.58
P08697	SERPINF2	Alpha-2-antiplasmin	92.05	9.18
A0A0C4DH25	IGKV3D-20	Immunoglobulin kappa variable 3D-20	92.05	6.28
P10643	C7	Complement component C7	92.05	4.08
P19022	CDH2	Cadherin-2	92.05	3.83
P19823	ITIH2	Inter-alpha-trypsin inhibitor heavy chain H2	90.91	10.06
Q5T3U5	ABCC10	Multidrug resistance-associated protein 7	90.91	5.73
P51693	APLP1	Amyloid-like protein 1	89.77	4.08
P07357	C8A	Complement component C8 alpha chain	89.77	3.88
P20273	CD22	B-cell receptor CD22	88.64	15.02
P98160	HSPG2	Basement membrane-specific heparan sulfate proteoglycan core protein	88.64	5.82
Q5T8P6	RBM26	RNA-binding protein 26	88.64	4.68
Q9Y287	ITM2B	Integral membrane protein 2B	88.64	3.43
P39060	COL18A1	Collagen alpha-1(XVIII) chain	88.64	2.36
P18065	IGFBP2	Insulin-like growth factor-binding protein 2	87.50	3.78
P10645	CHGA	Chromogranin-A	87.50	3.71
P05067	APP	Amyloid-beta precursor protein	86.36	10.08
P08294	SOD3	Extracellular superoxide dismutase [Cu-Zn]	86.36	5.86
Q92765	FRZB	Secreted frizzled-related protein 3	86.36	4.79
Q96S96	PEBP4	Phosphatidylethanolamine-binding protein 4	86.36	3.76
P01780	IGHV3-7	Immunoglobulin heavy variable 3-7	85.23	4.97
Q9HCJ0	TNRC6C	Trinucleotide repeat-containing gene 6C protein	85.23	3.70
O15240	VGF	Neurosecretory protein VGF	85.23	2.74
P02675	FGB	Fibrinogen beta chain	84.09	6.75
P55083	MFAP4	Microfibril-associated glycoprotein 4	84.09	2.81
P22914	CRYGS	Gamma-crystallin S	82.95	6.40
P00441	SOD1	Superoxide dismutase [Cu-Zn]	82.95	2.84
P34096	RNASE4	Ribonuclease 4	82.95	1.81
Q9Y6R7	FCGBP	IgGFc-binding protein	81.82	6.89
O75326	SEMA7A	Semaphorin-7A	81.82	5.49
P00736	C1R	Complement C1r subcomponent	81.82	4.49
Q9BZV3	IMPG2	Interphotoreceptor matrix proteoglycan 2	80.68	3.07
A0A087WSY6	IGKV3D-15	Immunoglobulin kappa variable 3D-15	80.68	2.17
P61626	LYZ	Lysozyme C	79.55	4.83
P43251	BTD	Biotinidase	79.55	2.71
P06733	ENO1	Alpha-enolase	78.41	12.05
P29622	SERPINA4	Kallistatin	78.41	5.03
P05546	SERPIND1	Heparin cofactor 2	78.41	5.03
P08185	SERPINA6	Corticosteroid-binding globulin	78.41	3.86
Q7Z7G0	ABI3BP	Target of Nesh-SH3	78.41	2.72
P04406	GAPDH	Glyceraldehyde-3-phosphate dehydrogenase	77.27	5.63
P01593	IGKV1D-33	Immunoglobulin kappa variable 1D-33	77.27	2.93
P02679	FGG	Fibrinogen gamma chain	76.14	4.92
Q66K66	TMEM198	Transmembrane protein 198	76.14	4.40
P02656	APOC3	Apolipoprotein C-III	76.14	3.19
P13671	C6	Complement component C6	76.14	2.96
Q99574	SERPINI1	Neuroserpin	76.14	2.38
P04264	KRT1	Keratin, type II cytoskeletal 1	75.00	36.86
Q9UHG2	PCSK1N	ProSAAS	75.00	3.28
P08571	CD14	Monocyte differentiation antigen CD14	75.00	2.48
Q86UD1	OAF	Out at first protein homolog	75.00	2.12
P98164	LRP2	Low-density lipoprotein receptor-related protein 2	75.00	1.84

**Table 3:** Five most abundant protein families in the human aqueous humor.

Family	Level	Proteins				
Apolipoproteins	High	APOA1	APOA2	APOA4	APOC3	APOD
		APOE	APOH			
	Medium					
	Low					
	Rare	APOB	APOC1	APOF	APOL1	APOLD1
Complement Proteins	High	C1R	C3	C4B	C6	C7
		C8A	C9	CFB	CFD	CFH
		CFI				
	Medium	C1S	C2	C4A	C5	C8B
		C8G				
	Low	C1QC	CFHR1	CFHR2		
	Rare	C1QB	C1QTNF6	C1QTNF7	C1RL	CD55
Immunoglobulins	High	IGHA1	IGHG2	IGHG3	IGHG4	IGHV3-7
		IGKC	IGKV1D-33	IGKV3D-15	IGKV3D-20	IGKV4-1
		IGLC2	IGLV1-47			
	Medium	IGHV3-49	IGHV5-51	IGHV6-1	IGKV1-8	IGKV2-28
		IGLV1-40	IGLV3-9			
	Low	IGHG1	IGHM	IGHV3-66	IGHV4-59	IGKV1-6
		IGKV1D-13	IGKV3D-11	IGLV6-57		
	Rare	IGDCC4	IGHA2	IGHV1-2	IGHV1-3	IGHV1-18
		IGHV1-46	IGHV1-69	IGHV2-26	IGHV2-70D	IGHV3-9
		IGHV3-15	IGHV3-64D	IGHV3-72	IGHV3-74	IGKV1-5
		IGKV1-16	IGKV1-17	IGKV1-27	IGKV3-11	IGKV3-20
		IGLV1-44	IGLV1-51	IGLV2-11	IGLV2-14	IGLV2-18
		IGLV3-10	IGLV3-19	IGLV3-21	IGSF10	IGSF21
		IGSF22	ILDR1	ISLR	JCHAIN	
Insulin Growth Factor (IGF) Family	High	IGFBP2	IGFBP6	IGFBP7		
	Medium	IGF2	IGFBP5			
	Low	IGFALS	IGFBP3			
	Rare	IGF1R	IGF2BP1	IGFBP4		
Serine Protease Inhibitors (SERPINs)	High	SERPINA1	SERPINA3	SERPINA4	SERPINA6	SERPINC1
		SERPIND1	SERPINF1	SERPINF2	SERPING1	SERPINI1
	Medium	SERPINA5	SERPINA7			
	Low					
	Rare	SERPINB3	SERPINB13	SERPINE3	SERPINH1	

**Table 4:** Canonical pathways enriched in the constitutive aqueous humor proteome.

Canonical Pathway	*p*-Value	# of Proteins
Acute Phase Response Signaling	5.01 × 10^−42^	40
LXR/RXR Activation	5.01 × 10^−38^	33
FXR/RXR Activation	1.00 × 10^−35^	32
Complement System	1.00 × 10^−24^	17
Coagulation System	2.00 × 10^−19^	14
Clathrin-mediated Endocytosis Signaling	1.58 × 10^−12^	18
Atherosclerosis Signaling	3.98 × 10^−12^	15
IL-12 Signaling and Production in Macrophages	1.00 × 10^−10^	14
Extrinsic Prothrombin Activation Pathway	1.15 × 10^−10^	7
Intrinsic Prothrombin Activation Pathway	3.55 × 10^−10^	9
Production of Nitric Oxide and Reactive Oxygen Species in Macrophages	1.02 × 10^−8^	14
Hepatic Fibrosis/Hepatic Stellate Cell Activation	6.92 × 10^−8^	13
Maturity Onset Diabetes of Young (MODY) Signaling	1.74 × 10^−7^	8
Airway Pathology in Chronic Obstructive Pulmonary Disease	3.63 × 10^−6^	9
Systemic Lupus Erythematosus Signaling	4.68 × 10^−6^	12
Neuroprotective Role of THOP1 in Alzheimer’s Disease	2.63 × 10^−5^	8
GP6 Signaling Pathway	2.29 × 10^−4^	7
Iron homeostasis signaling pathway	5.37 × 10^−4^	7
Actin Cytoskeleton Signaling	0.002	8
Role of Macrophages, Fibroblasts and Endothelial Cells in Rheumatoid Arthritis	0.016	8
Glucocorticoid Receptor Signaling	0.021	10

**Table 5:** Proteins with significant differences between African American and Caucasian subjects.

UniProt ID	Gene Symbol	Description	Fold Change	Adj. *p*-Value	Pathway
Upregulated in African American subjects					
P01593	IGKV1D-33	Immunoglobulin kappa variable 1D-33	2.191	0.024	Complement activation
P08294	SOD3	Extracellular superoxide dismutase [Cu-Zn]	2.190	0.017	Antioxidant, Heparin binding
P00736	C1R	Complement C1r subcomponent	2.182	0.014	Complement activation, classical pathway
P08603	CFH	Complement factor H	1.865	0.021	Complement activation, alternative pathway
P01023	A2M	Alpha-2-macroglobulin	1.489	0.047	Blood coagulation
P01024	C3	Complement C3	1.289	0.047	Endopeptidase inhibitor activity
Downregulated in African American subjects					
Q8NG11	TSPAN14	Tetraspanin-14	−2.089	0.005	Cellular protein metabolic process
P02753	RBP4	Retinol-binding protein 4	−1.753	0.002	Retinal and retinol binding
P02766	TTR	Transthyretin	−1.751	<0.001	Hormone activity
P07998	RNASE1	Ribonuclease pancreatic	−1.636	0.002	Nucleic acid binding
P41222	PTGDS	Prostaglandin-H2 D-isomerase	−1.435	0.002	Fatty acid binding

## Supplementary Materials

The following are available online at https://www.mdpi.com/2227-7382/8/4/34/s1, Table S1: Complete list of proteins detected in 88 aqueous humor samples.
